# Crystal structures of methyl 3-(4-iso­propyl­phen­yl)-1-methyl-1,2,3,3a,4,9b-hexa­hydro­thio­chromeno[4,3-*b*]pyrrole-3a-carboxyl­ate, methyl 1-methyl-3-(*o*-tol­yl)-1,2,3,3a,4,9b-hexa­hydro­thio­chromeno[4,3-*b*]pyrrole-3a-carboxyl­ate and methyl 1-methyl-3-(*o*-tol­yl)-3,3a,4,9b-tetra­hydro-1*H*-thio­chromeno[4,3-*c*]isoxazole-3a-carboxyl­ate

**DOI:** 10.1107/S2056989015008063

**Published:** 2015-05-07

**Authors:** R. Raja, M. Suresh, R. Raghunathan, A. SubbiahPandi

**Affiliations:** a Department of Physics, Presidency College (Autonomous), Chennai 600 005, India; b Department of Organic Chemistry, University of Madras, Guindy Campus, Chennai 602 025, India

**Keywords:** crystal structure, thio­chromene, isoxazole, pyrrole, chromeno­pyrrole, thio­pyran, C—H⋯π inter­actions

## Abstract

In the title compounds, C_23_H_27_NO_2_S, (I), and C_21_H_23_NO_2_S, (II), the pyrrole rings have envelope conformations with the C atom substituted by the benzene ring as the flap. In the third title compound, C_20_H_21_NO_3_S, (III), the isoxazole ring has a twisted conformation on the C—C bond substituted by the benzene ring and the carboxyl­ate group. In all three compounds, the thio­pyran ring has a half-chair conformation. The mean plane of the pyrrole ring is inclined to the mean plane of the thio­pyran ring by 57.07 (9), 58.98 (9) and 60.34 (12)° in (I), (II) and (III), respectively. The benzene rings are inclined to one another by 73.26 (10)° in (I), 65.781)° in (II) and 63.37 (13)° in (III). In the crystals of all three compounds, there are no classical hydrogen bonds present. Only in the crystal of compound (I) are mol­ecules linked by a pair of C—H⋯π inter­actions, forming inversion dimers. The isopropyl group in compound (I) is disordered over two sets of sites and has a refined occupancy ratio of 0.586 (13):0.414 (13).

## Abstract 2

Three thio­chromeno[4,3-*b*]pyrrole esters have very similar conformations. Structurally two of the compounds differ only by the substituent on the benzene ring, *i.e.* 4-iso­propyl­phenyl and *o*-tolyl, while two of the compounds differ only in that one has a pyrrole ring and one has an isoxazole ring.

## Chemical context   

Pyrrole derivatives are of considerable synthetic importance due to their extensive use in drug discovery (Toja *et al.*, 1987 ▸) which is linked to their pharmacological activity such as anti-inflammatory (Muchowski *et al.*, 1985 ▸), cytotoxicity (Dannhardt *et al.*, 2000 ▸) and their use in the treatment of hyper­lipidemias (Holub *et al.*, 2004 ▸) and as anti­tumour agents (Krowicki *et al.*, 1988 ▸). Other pyrrole-containing heterocyclic compounds have been reported previously for biological studies (Almerico *et al.*, 1998 ▸). Pyrrole derivatives have biological activity such as COX-1/COX-2 inhibitors (Dannhardt *et al.*, 2000 ▸) as well as cytotoxic activity against a variety of marine and human tumour models (Evans *et al.*, 2003 ▸). Isoxazoline derivatives have been shown to be efficient precursors for the preparation of many synthetic inter­mediates including γ-amino alcohols and β-hy­droxy ketones (Kozikowski, 1984 ▸). They display inter­esting biological properties such as herbicidal, plant-growth regulatory and anti­tumour activities (Howe & Shelton, 1990 ▸). Chromeno­pyrrole compounds are used in the treatment of impulsive disorders (Caine & Koob, 1993 ▸). Continuing our inter­est in such compounds, we have synthesized the title compounds and report herein on their crystal structures.
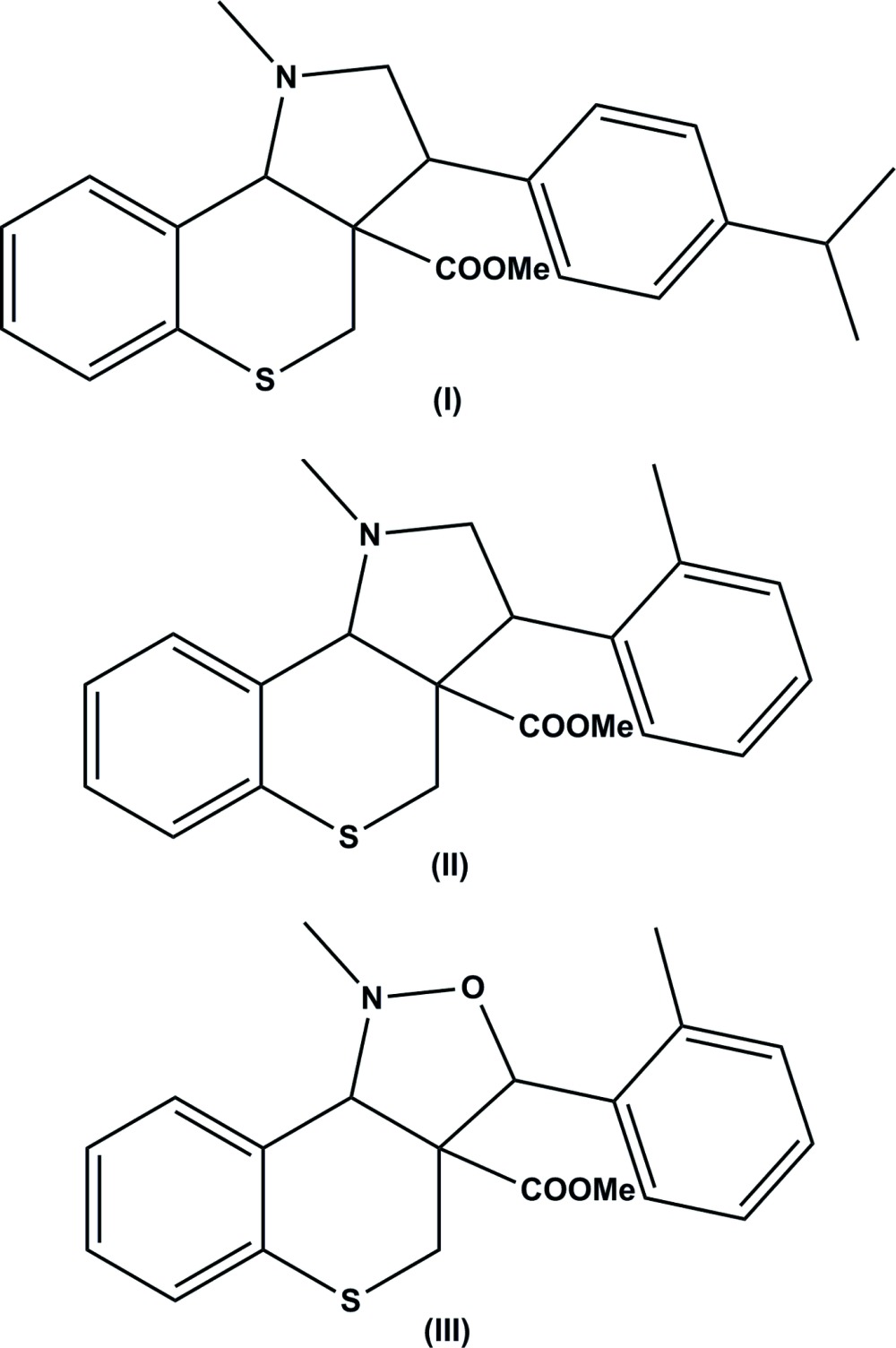


## Structural commentary   

The title compounds (I) and (II) differ only by the substituent on the benzene ring; 4-iso­propyl­phenyl in (I) and *o*-tolyl in (II). Compounds (II) and (III) differ only in that (II) has a pyrrole ring while (III) has an isoxazole ring.

**Figure 1:**
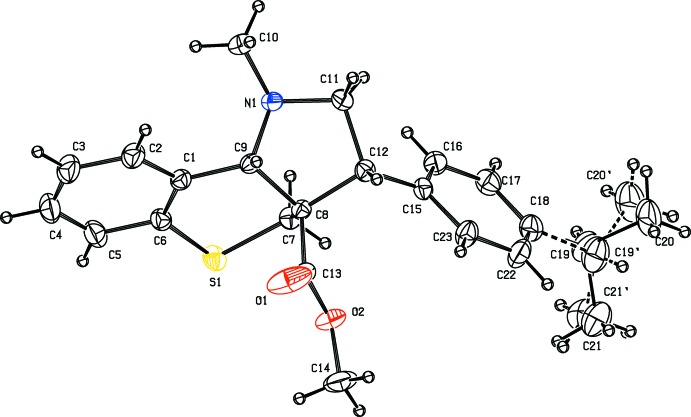
The mol­ecular structure of compound (I), showing the atom labelling. Displacement ellipsoids are drawn at the 30% probability level.

The mol­ecular structure of compound (I) is shown in Fig. 1 ▸. The five-membered methyl-substituted pyrrole ring adopts an envelope conformation with atom C9 as the flap, deviating from the mean plane defined by the plane of the other ring atoms by 0.0167 Å. The puckering parameters of this ring are *q*_2_ = 0.4713 (15) Å and φ_2_ = 41.27 (19)°. The thio­pyran ring has a half-chair conformation, with the lowest asymmetry parameters ΔC2(S1—C7) = 8.34 (16) Å. The mean plane of the pyrrole ring makes dihedral angles of 57.07 (9) and 63.29 (10)° with the mean plane of the thio­pyran ring and the benzene ring, respectively.

**Figure 2:**
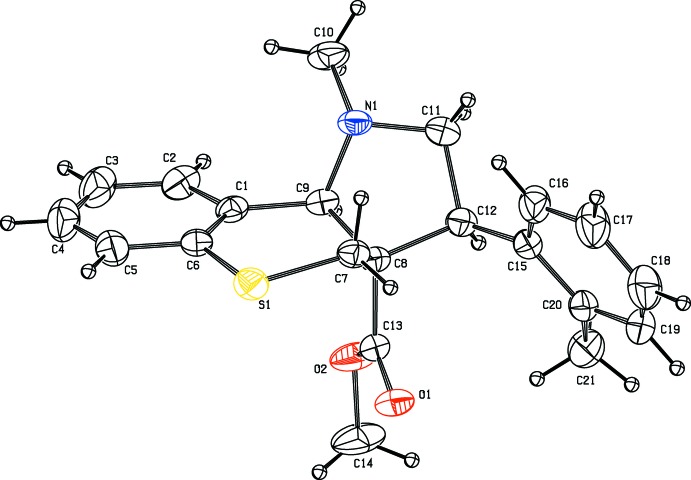
The mol­ecular structure of compound (II), showing the atom labelling. Displacement ellipsoids are drawn at the 30% probability level.

The mol­ecular structure of the compound (II) is illustrated in Fig. 2 ▸. The bond lengths and bond angles are similar to those in compound (I). The pyrrole ring (N1/C8–C12) adopts an envelope conformation with atom C9 atom as the flap having asymmetry parameters (Nardelli, 1983 ▸) ΔCS(C9) = 4.51 Å and with puckering parameters *q*_2_ = 0.4673 (18) Å, φ_2_ = 223.5 (2)°. As in (I), the thio­pyran ring has a half-chair conformation. The mean plane of the pyrrole ring is inclined to thio­pyran ring mean plane and the benzene ring by 58.98 (9) and 67.75 (11)°, respectively. The carboxyl­ate group assumes an extended conformation, as can be seen from the C8—C13—O2—C14 torsion angle of 175.4 (2)°.

**Figure 3:**
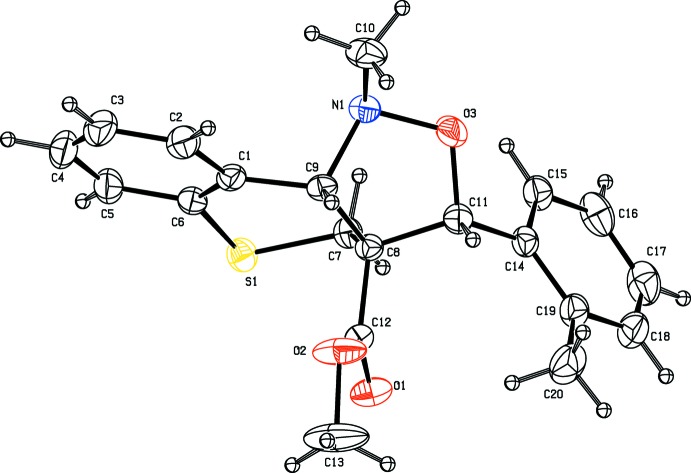
The mol­ecular structure of compound (III), showing the atom labelling. Displacement ellipsoids are drawn at the 30% probability level.

The mol­ecular structure of mol­ecule (III) is shown in Fig. 3 ▸. The isoxazole ring (N1/O3/C11/C8/C9) has a twist conformation about bond C9–C8: puckering parameters *q*_2_ = 0.466 (2) Å, φ_2_ = 275.7 (3)°. As in (I) and (II), the thio­pyran ring has a half-chair conformation. The dihedral angles between the mean plane of the isoxazole ring and the thio­pyran ring mean plane and the benzene ring are 60.34 (12) and 61.30 (14)°, respectively. The geometric parameters of mol­ecule (III) agree well with those reported for (I) and (II), and a closely related structure, 1-methyl-3-(naphthalen-1-yl)-3,3a,4,9b-tetra­hydro-1*H*-chromeno[4,3-*c*]isoxazole-3a-carbonitrile (Gangadharan *et al.*, 2011 ▸).

## Supra­molecular features   

**Figure 4:**
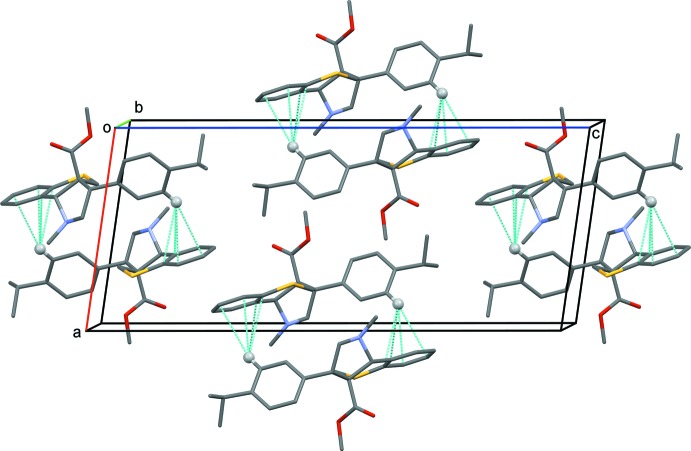
A view along the *b* axis of the crystal packing of compound (I). The dashed cyan lines represent the C—H⋯centroid distances (see Table 1 ▸).

In the crystals of compounds (I), (II) and (III), there are no classical hydrogen bonds present. Only in compound (I) is there a C—H⋯π inter­action present, and mol­ecules are linked by a pair of these inter­actions forming inversion dimers (Table 1 ▸ and Fig. 4 ▸).

## Database survey   

While a search of the Cambridge Structural Database (CSD, Version 5.36, November 2014; Groom & Allen, 2014 ▸) for chromenoisoxazole derivatives revealed over 30 hits, there were no hits for thio­chromeno­pyrroles or thio­chromenoisoxazoles.

## Synthesis and crystallization   

Compound (I): To a solution of methyl (*E*)-2-{[(2-formyl­phen­yl)thio]­meth­yl}-3-phenyl­acrylate (1 mmol) and sarcosine (1.2 mmol) in aceto­nitrile (10 ml), was added pyridine (0.2 mmol) and the mixture was refluxed until completion of the reaction (monitored by TLC). The crude product was subjected to column chromatography on silica gel (100–200 mesh) using petroleum ether–ethyl acetate (9:1) as eluent, which successfully provided the pure product as a colourless solid. The product was dissolved in chloro­form and heated for 2 min. The resulting solution were allowed to evaporate slowly at room temperature and yielded colourless block-like crystals of compound (I).

Compound (II): Here methyl (*E*)-2-{[(2-formyl­phen­yl)thio]­meth­yl}-3-(*o*-tol­yl) acrylate (1 mmol) and sarcosine (1.2 mmol) in aceto­nitrile (10 ml) were reacted with pyridine following the same procedure as for compound (I), and colourless crystals of compound (II) were obtained.

Compound (III): Here methyl (*E*)-2-{[(2-formyl­phen­yl)thio]­meth­yl}-3-(*o*-tol­yl) acrylate(1 mmol) and *N*-methyl hydroxyl­amine hydro­chloride (1.1 mmol) in aceto­nitrile (10 ml) were reacted with pyridine following the same procedure as for compounds (I) and (II), and colourless crystals of compound (III) were obtained.

## Refinement   

Crystal data, data collection and structure refinement details are summarized in Table 2 ▸. The C-bound H atoms were positioned geometrically (C—H = 0.93–0.98 Å) and allowed to ride on their parent atoms, with *U*_iso_(H) = 1.5*U*_eq_(C) for methyl H atoms and 1.2*U*_eq_(C) for other H atoms. The isopropyl group in compound (I), atoms C19–C21, is disordered over two sets of sites and has a refined occupancy ratio of 0.586 (13):0.414 (13).

## Supplementary Material

Crystal structure: contains datablock(s) global, I, II, III. DOI: 10.1107/S2056989015008063/su5106sup1.cif

Structure factors: contains datablock(s) I. DOI: 10.1107/S2056989015008063/su5106Isup2.hkl

Structure factors: contains datablock(s) II. DOI: 10.1107/S2056989015008063/su5106IIsup3.hkl

Structure factors: contains datablock(s) III. DOI: 10.1107/S2056989015008063/su5106IIIsup4.hkl

Supporting information file. DOI: 10.1107/S2056989015008063/su5106Isup5.cml

Supporting information file. DOI: 10.1107/S2056989015008063/su5106IIsup6.cml

Supporting information file. DOI: 10.1107/S2056989015008063/su5106IIIsup7.cml

CCDC references: 1061279, 1061278, 1061277

Additional supporting information:  crystallographic information; 3D view; checkCIF report

## Figures and Tables

**Table 1:** Hydrogen-bond geometry (Å, °) for (I) *Cg*3 is the centroid of the C1–C6 ring.

*D*—H⋯*A*	*D*—H	H⋯*A*	*D*⋯*A*	*D*—H⋯*A*
C17—H17⋯*Cg*3^i^	0.93	2.91	3.695 (2)	143

Symmetry code: (i) 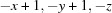.

**Table 2:** Experimental details

	(I)	(II)	(III)
Crystal data			
Chemical formula	C_23_H_27_NO_2_S	C_21_H_21_NO_2_S	C_20_H_21_NO_3_S
*M*_r_	381.52	351.45	355.44
Crystal system, space group	Monoclinic, *P*2_1_/*n*	Triclinic, *P* 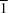	Orthorhombic, *P* *b* *c* *a*
Temperature (K)	293	293	293
*a*, *b*, *c* (Å)	10.7330 (3), 7.7568 (2), 24.9436 (7)	8.1882 (3), 10.4987 (4), 10.9594 (4)	11.2629 (11), 13.2117 (11), 24.041 (3)
α, β, γ (°)	90, 98.485 (1), 90	104.554 (1), 90.983 (1), 90.134 (1)	90, 90, 90
*V* (Å^3^)	2053.92 (10)	911.74 (6)	3577.3 (6)
*Z*	4	2	8
Radiation type	Mo *K*α	Mo *K*α	Mo *K*α
μ (mm^−1^)	0.18	0.19	0.20
Crystal size (mm)	0.35 × 0.30 × 0.25	0.35 × 0.30 × 0.25	0.35 × 0.30 × 0.25

Data collection			
Diffractometer	Bruker *SMART* APEXII CCD	Bruker *SMART* APEXII CCD	Bruker *SMART* APEXII CCD
Absorption correction	Multi-scan (*SADABS*; Bruker, 2008 ▸)	Multi-scan (*SADABS*; Bruker, 2008 ▸)	Multi-scan (*SADABS*; Bruker, 2008 ▸)
*T*_min_, *T*_max_	0.941, 0.958	0.935, 0.953	0.932, 0.951
No. of measured, independent and observed [*I* > 2σ(*I*)] reflections	16824, 3616, 3170	19010, 3210, 2790	37913, 3151, 2536
*R*_int_	0.019	0.020	0.033
(sin θ/λ)_max_ (Å^−1^)	0.595	0.595	0.595

Refinement			
*R*[*F* ^2^ > 2σ(*F* ^2^)], *wR*(*F* ^2^), *S*	0.041, 0.111, 1.06	0.038, 0.115, 1.07	0.046, 0.111, 1.12
No. of reflections	3616	3210	3151
No. of parameters	272	229	229
No. of restraints	107	0	0
H-atom treatment	H-atom parameters constrained	H-atom parameters constrained	H-atom parameters constrained
Δρ_max_, Δρ_min_ (e Å^−3^)	0.24, −0.27	0.26, −0.32	0.22, −0.22

Computer programs: *APEX2* and *SAINT* (Bruker, 2008 ▸), *SHELXS97* and *SHELXL97* (Sheldrick, 2008 ▸), *ORTEP-3* for Windows (Farrugia, 2012 ▸), *Mercury* (Macrae *et al.*, 2008 ▸) and *PLATON* (Spek, 2009 ▸).

## supplementary crystallographic information

### (I) Methyl 3-(4-isopropylphenyl)-1-methyl-1,2,3,3a,4,9b-hexahydrothiochromeno[4,3-b]pyrrole-3a-carboxylate Crystal data

**Table:**

C_23_H_27_NO_2_S	*F*(000) = 816
*M**_r_* = 381.52	*D*_x_ = 1.234 Mg m^−^^3^
Monoclinic, *P*2_1_/*n*	Mo *K*α radiation, λ = 0.71073 Å
Hall symbol: -P 2yn	Cell parameters from 3170 reflections
*a* = 10.7330 (3) Å	θ = 1.7–25.0°
*b* = 7.7568 (2) Å	µ = 0.18 mm^−^^1^
*c* = 24.9436 (7) Å	*T* = 293 K
β = 98.485 (1)°	Block, colourless
*V* = 2053.92 (10) Å^3^	0.35 × 0.30 × 0.25 mm
*Z* = 4	

### (I) Methyl 3-(4-isopropylphenyl)-1-methyl-1,2,3,3a,4,9b-hexahydrothiochromeno[4,3-b]pyrrole-3a-carboxylate Data collection

**Table:**

Bruker SMART APEXII CCD diffractometer	3616 independent reflections
Radiation source: fine-focus sealed tube	3170 reflections with *I* > 2σ(*I*)
Graphite monochromator	*R*_int_ = 0.019
ω and φ scans	θ_max_ = 25.0°, θ_min_ = 1.7°
Absorption correction: multi-scan (*SADABS*; Bruker, 2008)	*h* = −12→12
*T*_min_ = 0.941, *T*_max_ = 0.958	*k* = −9→9
16824 measured reflections	*l* = −24→29

### (I) Methyl 3-(4-isopropylphenyl)-1-methyl-1,2,3,3a,4,9b-hexahydrothiochromeno[4,3-b]pyrrole-3a-carboxylate Refinement

**Table:**

Refinement on *F*^2^	Primary atom site location: structure-invariant direct methods
Least-squares matrix: full	Secondary atom site location: difference Fourier map
*R*[*F*^2^ > 2σ(*F*^2^)] = 0.041	Hydrogen site location: inferred from neighbouring sites
*wR*(*F*^2^) = 0.111	H-atom parameters constrained
*S* = 1.06	*w* = 1/[σ^2^(*F*_o_^2^) + (0.050*P*)^2^ + 1.0371*P*] where *P* = (*F*_o_^2^ + 2*F*_c_^2^)/3
3616 reflections	(Δ/σ)_max_ < 0.001
272 parameters	Δρ_max_ = 0.24 e Å^−^^3^
107 restraints	Δρ_min_ = −0.27 e Å^−^^3^

### (I) Methyl 3-(4-isopropylphenyl)-1-methyl-1,2,3,3a,4,9b-hexahydrothiochromeno[4,3-b]pyrrole-3a-carboxylate Special details

**Table:**

Geometry. All e.s.d.'s (except the e.s.d. in the dihedral angle between two l.s. planes) are estimated using the full covariance matrix. The cell e.s.d.'s are taken into account individually in the estimation of e.s.d.'s in distances, angles and torsion angles; correlations between e.s.d.'s in cell parameters are only used when they are defined by crystal symmetry. An approximate (isotropic) treatment of cell e.s.d.'s is used for estimating e.s.d.'s involving l.s. planes.
Refinement. Refinement of *F*^2^ against ALL reflections. The weighted *R*-factor *wR* and goodness of fit *S* are based on *F*^2^, conventional *R*-factors *R* are based on *F*, with *F* set to zero for negative *F*^2^. The threshold expression of *F*^2^ > σ(*F*^2^) is used only for calculating *R*-factors(gt) *etc*. and is not relevant to the choice of reflections for refinement. *R*-factors based on *F*^2^ are statistically about twice as large as those based on *F*, and *R*- factors based on ALL data will be even larger.

### (I) Methyl 3-(4-isopropylphenyl)-1-methyl-1,2,3,3a,4,9b-hexahydrothiochromeno[4,3-b]pyrrole-3a-carboxylate Fractional atomic coordinates and isotropic or equivalent isotropic displacement parameters (Å^2^)

**Table:**

	*x*	*y*	*z*	*U*_iso_*/*U*_eq_	Occ. (<1)
C1	0.64364 (15)	0.4800 (2)	0.14949 (7)	0.0333 (4)	
C2	0.60663 (18)	0.4940 (3)	0.20060 (8)	0.0468 (5)	
H2	0.5834	0.6015	0.2124	0.056*	
C3	0.6033 (2)	0.3533 (3)	0.23429 (9)	0.0584 (6)	
H3	0.5765	0.3660	0.2679	0.070*	
C4	0.6398 (2)	0.1946 (3)	0.21793 (9)	0.0606 (6)	
H4	0.6379	0.0994	0.2405	0.073*	
C5	0.67929 (19)	0.1762 (3)	0.16824 (9)	0.0511 (5)	
H5	0.7052	0.0687	0.1576	0.061*	
C6	0.68086 (16)	0.3175 (2)	0.13353 (7)	0.0361 (4)	
C7	0.70294 (16)	0.4766 (2)	0.03604 (7)	0.0327 (4)	
H7A	0.7507	0.4820	0.0060	0.039*	
H7B	0.6142	0.4775	0.0212	0.039*	
C8	0.73260 (14)	0.6355 (2)	0.07149 (6)	0.0279 (4)	
C9	0.64648 (14)	0.6410 (2)	0.11561 (7)	0.0301 (4)	
H9	0.6724	0.7380	0.1399	0.036*	
C10	0.42438 (19)	0.7312 (3)	0.11351 (10)	0.0568 (6)	
H10A	0.4083	0.6368	0.1364	0.085*	
H10B	0.3492	0.7573	0.0889	0.085*	
H10C	0.4495	0.8305	0.1354	0.085*	
C11	0.55213 (17)	0.8256 (2)	0.04721 (8)	0.0399 (4)	
H11A	0.5384	0.9360	0.0636	0.048*	
H11B	0.4980	0.8183	0.0125	0.048*	
C12	0.69127 (16)	0.8060 (2)	0.03967 (7)	0.0324 (4)	
H12	0.7370	0.9003	0.0600	0.039*	
C13	0.87070 (15)	0.6497 (2)	0.09487 (7)	0.0347 (4)	
C14	1.07866 (18)	0.6040 (4)	0.07703 (11)	0.0687 (7)	
H14A	1.1210	0.5552	0.0493	0.103*	
H14B	1.1034	0.5433	0.1105	0.103*	
H14C	1.1010	0.7234	0.0818	0.103*	
C15	0.72199 (16)	0.8169 (2)	−0.01743 (7)	0.0333 (4)	
C16	0.64436 (17)	0.7505 (2)	−0.06219 (7)	0.0420 (4)	
H16	0.5687	0.6984	−0.0575	0.050*	
C17	0.67758 (19)	0.7603 (3)	−0.11355 (8)	0.0486 (5)	
H17	0.6232	0.7158	−0.1428	0.058*	
C18	0.7893 (2)	0.8346 (3)	−0.12255 (8)	0.0515 (5)	
C19	0.8153 (7)	0.8254 (11)	−0.1815 (2)	0.0583 (18)	0.586 (13)
H19	0.7602	0.7432	−0.2035	0.070*	0.586 (13)
C20	0.8006 (10)	1.0118 (12)	−0.2041 (3)	0.102 (3)	0.586 (13)
H20A	0.7144	1.0475	−0.2061	0.153*	0.586 (13)
H20B	0.8248	1.0152	−0.2396	0.153*	0.586 (13)
H20C	0.8536	1.0882	−0.1805	0.153*	0.586 (13)
C21	0.9546 (11)	0.7867 (18)	−0.1814 (5)	0.091 (3)	0.586 (13)
H21A	0.9717	0.7809	−0.2181	0.137*	0.586 (13)
H21B	0.9752	0.6784	−0.1637	0.137*	0.586 (13)
H21C	1.0044	0.8765	−0.1624	0.137*	0.586 (13)
C19'	0.8449 (11)	0.8718 (19)	−0.1752 (3)	0.073 (3)	0.414 (13)
H19'	0.8974	0.9752	−0.1685	0.087*	0.414 (13)
C20'	0.7403 (12)	0.915 (2)	−0.2199 (4)	0.104 (4)	0.414 (13)
H20D	0.6884	1.0039	−0.2079	0.156*	0.414 (13)
H20E	0.6902	0.8143	−0.2295	0.156*	0.414 (13)
H20F	0.7753	0.9552	−0.2509	0.156*	0.414 (13)
C21'	0.9299 (17)	0.728 (2)	−0.1893 (8)	0.088 (4)	0.414 (13)
H21D	0.9944	0.7061	−0.1591	0.132*	0.414 (13)
H21E	0.9681	0.7605	−0.2203	0.132*	0.414 (13)
H21F	0.8810	0.6248	−0.1976	0.132*	0.414 (13)
C22	0.8668 (2)	0.9020 (3)	−0.07806 (9)	0.0568 (6)	
H22	0.9427	0.9532	−0.0829	0.068*	
C23	0.83327 (18)	0.8944 (3)	−0.02667 (8)	0.0462 (5)	
H23	0.8865	0.9422	0.0023	0.055*	
N1	0.52473 (13)	0.68409 (19)	0.08280 (6)	0.0361 (3)	
O1	0.90964 (14)	0.7164 (3)	0.13667 (7)	0.0840 (7)	
O2	0.94460 (11)	0.5889 (2)	0.06139 (6)	0.0505 (4)	
S1	0.73931 (5)	0.27778 (6)	0.07238 (2)	0.04255 (16)	

### (I) Methyl 3-(4-isopropylphenyl)-1-methyl-1,2,3,3a,4,9b-hexahydrothiochromeno[4,3-b]pyrrole-3a-carboxylate Atomic displacement parameters (Å^2^)

**Table:**

	*U*^11^	*U*^22^	*U*^33^	*U*^12^	*U*^13^	*U*^23^
C1	0.0291 (8)	0.0381 (9)	0.0328 (9)	−0.0026 (7)	0.0045 (7)	0.0019 (7)
C2	0.0467 (11)	0.0570 (12)	0.0382 (10)	−0.0011 (9)	0.0113 (8)	0.0033 (9)
C3	0.0558 (12)	0.0821 (17)	0.0382 (11)	−0.0046 (12)	0.0099 (9)	0.0161 (11)
C4	0.0603 (13)	0.0663 (15)	0.0530 (13)	−0.0048 (11)	0.0011 (10)	0.0293 (12)
C5	0.0538 (12)	0.0402 (11)	0.0565 (13)	−0.0005 (9)	−0.0007 (10)	0.0140 (9)
C6	0.0327 (9)	0.0353 (9)	0.0387 (10)	−0.0027 (7)	0.0000 (7)	0.0054 (8)
C7	0.0379 (9)	0.0283 (8)	0.0321 (9)	−0.0001 (7)	0.0060 (7)	−0.0029 (7)
C8	0.0293 (8)	0.0258 (8)	0.0297 (8)	0.0000 (6)	0.0075 (6)	−0.0021 (7)
C9	0.0293 (8)	0.0287 (8)	0.0336 (9)	−0.0008 (7)	0.0087 (7)	−0.0037 (7)
C10	0.0387 (10)	0.0608 (13)	0.0763 (15)	0.0117 (10)	0.0258 (10)	0.0141 (12)
C11	0.0406 (10)	0.0338 (9)	0.0469 (11)	0.0094 (8)	0.0122 (8)	0.0075 (8)
C12	0.0366 (9)	0.0247 (8)	0.0367 (9)	−0.0019 (7)	0.0079 (7)	−0.0007 (7)
C13	0.0316 (9)	0.0373 (9)	0.0362 (10)	−0.0008 (7)	0.0086 (7)	−0.0024 (8)
C14	0.0303 (10)	0.0772 (17)	0.1015 (19)	0.0028 (11)	0.0199 (11)	−0.0011 (15)
C15	0.0360 (9)	0.0279 (8)	0.0361 (9)	−0.0006 (7)	0.0055 (7)	0.0050 (7)
C16	0.0374 (9)	0.0444 (11)	0.0431 (11)	−0.0042 (8)	0.0022 (8)	0.0043 (8)
C17	0.0507 (11)	0.0549 (12)	0.0377 (11)	0.0014 (10)	−0.0015 (9)	0.0009 (9)
C18	0.0574 (12)	0.0605 (13)	0.0378 (11)	0.0086 (10)	0.0110 (9)	0.0093 (10)
C19	0.056 (3)	0.082 (4)	0.037 (3)	−0.016 (3)	0.010 (2)	−0.003 (2)
C20	0.128 (6)	0.122 (6)	0.063 (4)	0.034 (5)	0.039 (4)	0.048 (4)
C21	0.100 (6)	0.128 (8)	0.057 (5)	0.017 (5)	0.046 (5)	0.013 (5)
C19'	0.090 (6)	0.088 (6)	0.043 (4)	−0.009 (5)	0.018 (4)	0.001 (4)
C20'	0.127 (8)	0.132 (9)	0.053 (5)	0.016 (6)	0.012 (5)	0.031 (5)
C21'	0.085 (7)	0.122 (10)	0.058 (5)	0.015 (7)	0.016 (5)	−0.012 (6)
C22	0.0504 (12)	0.0700 (15)	0.0533 (13)	−0.0146 (11)	0.0186 (10)	0.0108 (11)
C23	0.0446 (10)	0.0521 (12)	0.0418 (11)	−0.0145 (9)	0.0059 (8)	0.0025 (9)
N1	0.0288 (7)	0.0338 (8)	0.0468 (9)	0.0040 (6)	0.0097 (6)	0.0054 (7)
O1	0.0382 (8)	0.1496 (19)	0.0630 (10)	−0.0111 (9)	0.0038 (7)	−0.0553 (12)
O2	0.0319 (6)	0.0652 (9)	0.0575 (8)	0.0022 (6)	0.0169 (6)	−0.0123 (7)
S1	0.0551 (3)	0.0263 (2)	0.0470 (3)	0.00570 (19)	0.0104 (2)	−0.00152 (19)

### (I) Methyl 3-(4-isopropylphenyl)-1-methyl-1,2,3,3a,4,9b-hexahydrothiochromeno[4,3-b]pyrrole-3a-carboxylate Geometric parameters (Å, º)

**Table:**

C1—C2	1.394 (2)	C14—H14A	0.9600
C1—C6	1.398 (2)	C14—H14B	0.9600
C1—C9	1.510 (2)	C14—H14C	0.9600
C2—C3	1.382 (3)	C15—C23	1.386 (2)
C2—H2	0.9300	C15—C16	1.390 (2)
C3—C4	1.372 (4)	C16—C17	1.382 (3)
C3—H3	0.9300	C16—H16	0.9300
C4—C5	1.375 (3)	C17—C18	1.378 (3)
C4—H4	0.9300	C17—H17	0.9300
C5—C6	1.398 (3)	C18—C22	1.387 (3)
C5—H5	0.9300	C18—C19	1.539 (5)
C6—S1	1.7599 (19)	C18—C19'	1.546 (7)
C7—C8	1.523 (2)	C19—C21	1.524 (12)
C7—S1	1.8024 (17)	C19—C20	1.551 (11)
C7—H7A	0.9700	C19—H19	0.9800
C7—H7B	0.9700	C20—H20A	0.9600
C8—C13	1.515 (2)	C20—H20B	0.9600
C8—C9	1.539 (2)	C20—H20C	0.9600
C8—C12	1.572 (2)	C21—H21A	0.9600
C9—N1	1.474 (2)	C21—H21B	0.9600
C9—H9	0.9800	C21—H21C	0.9600
C10—N1	1.457 (2)	C19'—C20'	1.500 (14)
C10—H10A	0.9600	C19'—C21'	1.519 (15)
C10—H10B	0.9600	C19'—H19'	0.9800
C10—H10C	0.9600	C20'—H20D	0.9600
C11—N1	1.468 (2)	C20'—H20E	0.9600
C11—C12	1.540 (2)	C20'—H20F	0.9600
C11—H11A	0.9700	C21'—H21D	0.9600
C11—H11B	0.9700	C21'—H21E	0.9600
C12—C15	1.511 (2)	C21'—H21F	0.9600
C12—H12	0.9800	C22—C23	1.383 (3)
C13—O1	1.184 (2)	C22—H22	0.9300
C13—O2	1.321 (2)	C23—H23	0.9300
C14—O2	1.439 (2)		
			
C2—C1—C6	117.55 (17)	C23—C15—C16	117.15 (16)
C2—C1—C9	118.63 (16)	C23—C15—C12	119.62 (15)
C6—C1—C9	123.77 (15)	C16—C15—C12	123.22 (15)
C3—C2—C1	122.1 (2)	C17—C16—C15	121.20 (17)
C3—C2—H2	119.0	C17—C16—H16	119.4
C1—C2—H2	119.0	C15—C16—H16	119.4
C4—C3—C2	119.6 (2)	C18—C17—C16	121.59 (18)
C4—C3—H3	120.2	C18—C17—H17	119.2
C2—C3—H3	120.2	C16—C17—H17	119.2
C3—C4—C5	120.0 (2)	C17—C18—C22	117.43 (18)
C3—C4—H4	120.0	C17—C18—C19	114.9 (3)
C5—C4—H4	120.0	C22—C18—C19	127.6 (3)
C4—C5—C6	120.7 (2)	C17—C18—C19'	132.0 (5)
C4—C5—H5	119.7	C22—C18—C19'	110.4 (5)
C6—C5—H5	119.7	C19—C18—C19'	18.3 (5)
C1—C6—C5	120.03 (18)	C21—C19—C18	108.9 (7)
C1—C6—S1	123.93 (13)	C21—C19—C20	103.4 (9)
C5—C6—S1	115.96 (15)	C18—C19—C20	106.3 (6)
C8—C7—S1	112.92 (11)	C21—C19—H19	112.6
C8—C7—H7A	109.0	C18—C19—H19	112.6
S1—C7—H7A	109.0	C20—C19—H19	112.6
C8—C7—H7B	109.0	C19—C20—H20A	109.5
S1—C7—H7B	109.0	C19—C20—H20B	109.5
H7A—C7—H7B	107.8	H20A—C20—H20B	109.5
C13—C8—C7	113.01 (13)	C19—C20—H20C	109.5
C13—C8—C9	112.32 (13)	H20A—C20—H20C	109.5
C7—C8—C9	109.95 (13)	H20B—C20—H20C	109.5
C13—C8—C12	108.71 (13)	C19—C21—H21A	109.5
C7—C8—C12	111.61 (13)	C19—C21—H21B	109.5
C9—C8—C12	100.59 (12)	H21A—C21—H21B	109.5
N1—C9—C1	114.12 (13)	C19—C21—H21C	109.5
N1—C9—C8	100.71 (13)	H21A—C21—H21C	109.5
C1—C9—C8	116.20 (13)	H21B—C21—H21C	109.5
N1—C9—H9	108.5	C20'—C19'—C21'	113.7 (14)
C1—C9—H9	108.5	C20'—C19'—C18	109.5 (8)
C8—C9—H9	108.5	C21'—C19'—C18	112.4 (12)
N1—C10—H10A	109.5	C20'—C19'—H19'	107.0
N1—C10—H10B	109.5	C21'—C19'—H19'	107.0
H10A—C10—H10B	109.5	C18—C19'—H19'	107.0
N1—C10—H10C	109.5	C19'—C20'—H20D	109.5
H10A—C10—H10C	109.5	C19'—C20'—H20E	109.5
H10B—C10—H10C	109.5	H20D—C20'—H20E	109.5
N1—C11—C12	106.52 (13)	C19'—C20'—H20F	109.5
N1—C11—H11A	110.4	H20D—C20'—H20F	109.5
C12—C11—H11A	110.4	H20E—C20'—H20F	109.5
N1—C11—H11B	110.4	C19'—C21'—H21D	109.5
C12—C11—H11B	110.4	C19'—C21'—H21E	109.5
H11A—C11—H11B	108.6	H21D—C21'—H21E	109.5
C15—C12—C11	117.38 (14)	C19'—C21'—H21F	109.5
C15—C12—C8	116.04 (13)	H21D—C21'—H21F	109.5
C11—C12—C8	103.18 (13)	H21E—C21'—H21F	109.5
C15—C12—H12	106.5	C23—C22—C18	121.21 (19)
C11—C12—H12	106.5	C23—C22—H22	119.4
C8—C12—H12	106.5	C18—C22—H22	119.4
O1—C13—O2	123.11 (16)	C22—C23—C15	121.41 (18)
O1—C13—C8	124.68 (16)	C22—C23—H23	119.3
O2—C13—C8	112.07 (14)	C15—C23—H23	119.3
O2—C14—H14A	109.5	C10—N1—C11	111.09 (15)
O2—C14—H14B	109.5	C10—N1—C9	115.36 (15)
H14A—C14—H14B	109.5	C11—N1—C9	105.37 (13)
O2—C14—H14C	109.5	C13—O2—C14	117.99 (16)
H14A—C14—H14C	109.5	C6—S1—C7	101.98 (8)
H14B—C14—H14C	109.5		
			
C6—C1—C2—C3	−1.5 (3)	C8—C12—C15—C23	−93.3 (2)
C9—C1—C2—C3	−179.07 (17)	C11—C12—C15—C16	−36.9 (2)
C1—C2—C3—C4	1.4 (3)	C8—C12—C15—C16	85.7 (2)
C2—C3—C4—C5	−0.1 (3)	C23—C15—C16—C17	0.5 (3)
C3—C4—C5—C6	−1.0 (3)	C12—C15—C16—C17	−178.64 (17)
C2—C1—C6—C5	0.4 (3)	C15—C16—C17—C18	0.7 (3)
C9—C1—C6—C5	177.81 (16)	C16—C17—C18—C22	−1.0 (3)
C2—C1—C6—S1	−176.06 (13)	C16—C17—C18—C19	176.5 (4)
C9—C1—C6—S1	1.3 (2)	C16—C17—C18—C19'	−175.7 (7)
C4—C5—C6—C1	0.8 (3)	C17—C18—C19—C21	−140.0 (8)
C4—C5—C6—S1	177.59 (16)	C22—C18—C19—C21	37.2 (10)
S1—C7—C8—C13	61.01 (16)	C19'—C18—C19—C21	58 (2)
S1—C7—C8—C9	−65.37 (15)	C17—C18—C19—C20	109.2 (7)
S1—C7—C8—C12	−176.10 (11)	C22—C18—C19—C20	−73.6 (8)
C2—C1—C9—N1	−86.08 (19)	C19'—C18—C19—C20	−52 (2)
C6—C1—C9—N1	96.55 (19)	C17—C18—C19'—C20'	33.7 (16)
C2—C1—C9—C8	157.34 (15)	C22—C18—C19'—C20'	−141.3 (12)
C6—C1—C9—C8	−20.0 (2)	C19—C18—C19'—C20'	57 (2)
C13—C8—C9—N1	161.82 (13)	C17—C18—C19'—C21'	−93.5 (12)
C7—C8—C9—N1	−71.42 (15)	C22—C18—C19'—C21'	91.4 (13)
C12—C8—C9—N1	46.38 (14)	C19—C18—C19'—C21'	−71 (2)
C13—C8—C9—C1	−74.36 (18)	C17—C18—C22—C23	0.2 (3)
C7—C8—C9—C1	52.41 (18)	C19—C18—C22—C23	−177.0 (4)
C12—C8—C9—C1	170.21 (13)	C19'—C18—C22—C23	176.0 (6)
N1—C11—C12—C15	132.25 (15)	C18—C22—C23—C15	1.0 (3)
N1—C11—C12—C8	3.27 (17)	C16—C15—C23—C22	−1.3 (3)
C13—C8—C12—C15	81.94 (17)	C12—C15—C23—C22	177.85 (19)
C7—C8—C12—C15	−43.37 (18)	C12—C11—N1—C10	152.28 (16)
C9—C8—C12—C15	−159.95 (14)	C12—C11—N1—C9	26.68 (18)
C13—C8—C12—C11	−148.26 (14)	C1—C9—N1—C10	65.7 (2)
C7—C8—C12—C11	86.43 (16)	C8—C9—N1—C10	−169.06 (15)
C9—C8—C12—C11	−30.15 (15)	C1—C9—N1—C11	−171.39 (14)
C7—C8—C13—O1	−150.6 (2)	C8—C9—N1—C11	−46.15 (16)
C9—C8—C13—O1	−25.5 (3)	O1—C13—O2—C14	0.5 (3)
C12—C8—C13—O1	84.9 (2)	C8—C13—O2—C14	176.23 (17)
C7—C8—C13—O2	33.7 (2)	C1—C6—S1—C7	−11.94 (17)
C9—C8—C13—O2	158.85 (15)	C5—C6—S1—C7	171.45 (14)
C12—C8—C13—O2	−90.74 (17)	C8—C7—S1—C6	43.36 (13)
C11—C12—C15—C23	144.07 (17)		

### (I) Methyl 3-(4-isopropylphenyl)-1-methyl-1,2,3,3a,4,9b-hexahydrothiochromeno[4,3-b]pyrrole-3a-carboxylate Hydrogen-bond geometry (Å, º)

Cg3 is the centroid of the C1–C6 ring.

**Table:**

*D*—H···*A*	*D*—H	H···*A*	*D*···*A*	*D*—H···*A*
C17—H17···*Cg*3^i^	0.93	2.91	3.695 (2)	143

Symmetry code: (i) −*x*+1, −*y*+1, −*z*.

### (II) Methyl 1-methyl-3-(o-tolyl)-1,2,3,3a,4,9b-hexahydrothiochromeno[4,3-b]pyrrole-3a-carboxylate Crystal data

**Table:**

C_21_H_21_NO_2_S	*Z* = 2
*M**_r_* = 351.45	*F*(000) = 372
Triclinic, *P*1	*D*_x_ = 1.280 Mg m^−^^3^
Hall symbol: -P 1	Mo *K*α radiation, λ = 0.71073 Å
*a* = 8.1882 (3) Å	Cell parameters from 2790 reflections
*b* = 10.4987 (4) Å	θ = 1.9–25.0°
*c* = 10.9594 (4) Å	µ = 0.19 mm^−^^1^
α = 104.554 (1)°	*T* = 293 K
β = 90.983 (1)°	Block, colourless
γ = 90.134 (1)°	0.35 × 0.30 × 0.25 mm
*V* = 911.74 (6) Å^3^	

### (II) Methyl 1-methyl-3-(o-tolyl)-1,2,3,3a,4,9b-hexahydrothiochromeno[4,3-b]pyrrole-3a-carboxylate Data collection

**Table:**

Bruker SMART APEXII CCD diffractometer	3210 independent reflections
Radiation source: fine-focus sealed tube	2790 reflections with *I* > 2σ(*I*)
Graphite monochromator	*R*_int_ = 0.020
ω and φ scans	θ_max_ = 25.0°, θ_min_ = 1.9°
Absorption correction: multi-scan (*SADABS*; Bruker, 2008)	*h* = −9→9
*T*_min_ = 0.935, *T*_max_ = 0.953	*k* = −12→12
19010 measured reflections	*l* = −13→13

### (II) Methyl 1-methyl-3-(o-tolyl)-1,2,3,3a,4,9b-hexahydrothiochromeno[4,3-b]pyrrole-3a-carboxylate Refinement

**Table:**

Refinement on *F*^2^	Primary atom site location: structure-invariant direct methods
Least-squares matrix: full	Secondary atom site location: difference Fourier map
*R*[*F*^2^ > 2σ(*F*^2^)] = 0.038	Hydrogen site location: inferred from neighbouring sites
*wR*(*F*^2^) = 0.115	H-atom parameters constrained
*S* = 1.07	*w* = 1/[σ^2^(*F*_o_^2^) + (0.0591*P*)^2^ + 0.2766*P*] where *P* = (*F*_o_^2^ + 2*F*_c_^2^)/3
3210 reflections	(Δ/σ)_max_ < 0.001
229 parameters	Δρ_max_ = 0.26 e Å^−^^3^
0 restraints	Δρ_min_ = −0.32 e Å^−^^3^

### (II) Methyl 1-methyl-3-(o-tolyl)-1,2,3,3a,4,9b-hexahydrothiochromeno[4,3-b]pyrrole-3a-carboxylate Special details

**Table:**

Geometry. All e.s.d.'s (except the e.s.d. in the dihedral angle between two l.s. planes) are estimated using the full covariance matrix. The cell e.s.d.'s are taken into account individually in the estimation of e.s.d.'s in distances, angles and torsion angles; correlations between e.s.d.'s in cell parameters are only used when they are defined by crystal symmetry. An approximate (isotropic) treatment of cell e.s.d.'s is used for estimating e.s.d.'s involving l.s. planes.
Refinement. Refinement of *F*^2^ against ALL reflections. The weighted *R*-factor *wR* and goodness of fit *S* are based on *F*^2^, conventional *R*-factors *R* are based on *F*, with *F* set to zero for negative *F*^2^. The threshold expression of *F*^2^ > σ(*F*^2^) is used only for calculating *R*-factors(gt) *etc*. and is not relevant to the choice of reflections for refinement. *R*-factors based on *F*^2^ are statistically about twice as large as those based on *F*, and *R*- factors based on ALL data will be even larger.

### (II) Methyl 1-methyl-3-(o-tolyl)-1,2,3,3a,4,9b-hexahydrothiochromeno[4,3-b]pyrrole-3a-carboxylate Fractional atomic coordinates and isotropic or equivalent isotropic displacement parameters (Å^2^)

**Table:**

	*x*	*y*	*z*	*U*_iso_*/*U*_eq_	
C1	0.1321 (2)	0.62046 (16)	0.33099 (15)	0.0424 (4)	
C2	0.0560 (3)	0.6916 (2)	0.43961 (18)	0.0589 (5)	
H2	−0.0253	0.6509	0.4748	0.071*	
C3	0.0976 (3)	0.8203 (2)	0.4965 (2)	0.0759 (7)	
H3	0.0434	0.8661	0.5679	0.091*	
C4	0.2199 (4)	0.8801 (2)	0.4468 (2)	0.0768 (7)	
H4	0.2487	0.9670	0.4845	0.092*	
C5	0.2994 (3)	0.81250 (19)	0.3421 (2)	0.0614 (5)	
H5	0.3836	0.8534	0.3102	0.074*	
C6	0.2560 (2)	0.68236 (17)	0.28217 (16)	0.0444 (4)	
C7	0.2650 (2)	0.45327 (17)	0.09015 (16)	0.0435 (4)	
H7A	0.1727	0.4687	0.0392	0.052*	
H7B	0.3373	0.3921	0.0356	0.052*	
C8	0.20327 (18)	0.39039 (15)	0.19172 (15)	0.0389 (4)	
C9	0.07923 (18)	0.48030 (16)	0.27409 (15)	0.0397 (4)	
H9	0.0487	0.4408	0.3426	0.048*	
C10	−0.2119 (2)	0.5208 (2)	0.2449 (2)	0.0627 (5)	
H10A	−0.3002	0.4990	0.1844	0.094*	
H10B	−0.2028	0.6148	0.2747	0.094*	
H10C	−0.2328	0.4835	0.3146	0.094*	
C11	−0.0773 (2)	0.3283 (2)	0.1264 (2)	0.0678 (6)	
H11A	−0.1131	0.3147	0.0392	0.081*	
H11B	−0.1569	0.2884	0.1702	0.081*	
C12	0.0921 (2)	0.26691 (17)	0.13364 (18)	0.0475 (4)	
H12	0.0856	0.2166	0.1976	0.057*	
C15	0.1522 (2)	0.17354 (16)	0.01548 (17)	0.0480 (4)	
C16	0.1305 (3)	0.2006 (2)	−0.10163 (19)	0.0648 (6)	
H14	0.0742	0.2760	−0.1067	0.078*	
C17	0.1898 (3)	0.1190 (2)	−0.2106 (2)	0.0800 (7)	
H15	0.1722	0.1387	−0.2878	0.096*	
C18	0.2745 (3)	0.0093 (2)	−0.2041 (2)	0.0790 (7)	
H16	0.3181	−0.0447	−0.2767	0.095*	
C19	0.2953 (3)	−0.02130 (19)	−0.0912 (2)	0.0669 (6)	
H17	0.3519	−0.0972	−0.0884	0.080*	
C20	0.2343 (2)	0.05785 (17)	0.02048 (19)	0.0520 (4)	
C21	0.2546 (3)	0.0131 (2)	0.1378 (2)	0.0727 (6)	
H19A	0.3050	−0.0719	0.1187	0.109*	
H19B	0.1495	0.0075	0.1738	0.109*	
H19C	0.3223	0.0748	0.1969	0.109*	
C13	0.3469 (2)	0.34856 (16)	0.26130 (16)	0.0431 (4)	
C14	0.4427 (3)	0.3058 (3)	0.4510 (2)	0.0920 (9)	
H21A	0.4697	0.2173	0.4077	0.138*	
H21B	0.4048	0.3074	0.5338	0.138*	
H21C	0.5379	0.3609	0.4579	0.138*	
N1	−0.06074 (15)	0.46851 (14)	0.18600 (14)	0.0455 (4)	
O1	0.47275 (15)	0.30893 (14)	0.21352 (13)	0.0592 (4)	
O2	0.31503 (18)	0.35417 (16)	0.38084 (13)	0.0709 (4)	
S1	0.37215 (5)	0.60605 (5)	0.15186 (5)	0.05272 (17)	

### (II) Methyl 1-methyl-3-(o-tolyl)-1,2,3,3a,4,9b-hexahydrothiochromeno[4,3-b]pyrrole-3a-carboxylate Atomic displacement parameters (Å^2^)

**Table:**

	*U*^11^	*U*^22^	*U*^33^	*U*^12^	*U*^13^	*U*^23^
C1	0.0429 (9)	0.0469 (9)	0.0392 (9)	0.0106 (7)	−0.0039 (7)	0.0146 (7)
C2	0.0667 (12)	0.0659 (12)	0.0451 (10)	0.0183 (10)	0.0044 (9)	0.0155 (9)
C3	0.1017 (18)	0.0676 (14)	0.0490 (12)	0.0260 (13)	−0.0033 (12)	−0.0027 (11)
C4	0.112 (2)	0.0520 (12)	0.0602 (14)	0.0051 (13)	−0.0200 (13)	0.0042 (10)
C5	0.0735 (13)	0.0514 (11)	0.0618 (12)	−0.0055 (10)	−0.0200 (10)	0.0202 (10)
C6	0.0452 (9)	0.0454 (9)	0.0445 (9)	0.0049 (7)	−0.0109 (7)	0.0158 (7)
C7	0.0355 (8)	0.0514 (10)	0.0436 (9)	0.0035 (7)	0.0040 (7)	0.0118 (7)
C8	0.0316 (8)	0.0413 (8)	0.0440 (9)	0.0044 (6)	−0.0002 (6)	0.0113 (7)
C9	0.0332 (8)	0.0478 (9)	0.0423 (9)	0.0069 (7)	0.0036 (6)	0.0187 (7)
C10	0.0335 (9)	0.0740 (13)	0.0857 (15)	0.0107 (9)	0.0090 (9)	0.0290 (11)
C11	0.0379 (10)	0.0607 (12)	0.0978 (16)	0.0003 (9)	−0.0111 (10)	0.0075 (11)
C12	0.0390 (9)	0.0453 (9)	0.0590 (11)	−0.0005 (7)	−0.0042 (8)	0.0148 (8)
C15	0.0458 (9)	0.0391 (9)	0.0579 (11)	−0.0027 (7)	−0.0109 (8)	0.0109 (8)
C16	0.0859 (15)	0.0476 (11)	0.0579 (12)	0.0018 (10)	−0.0216 (11)	0.0093 (9)
C17	0.114 (2)	0.0635 (14)	0.0567 (13)	−0.0073 (13)	−0.0173 (13)	0.0061 (11)
C18	0.0939 (18)	0.0674 (14)	0.0630 (14)	−0.0041 (12)	−0.0038 (12)	−0.0067 (11)
C19	0.0627 (13)	0.0398 (10)	0.0896 (16)	0.0022 (9)	−0.0081 (11)	0.0011 (10)
C20	0.0483 (10)	0.0390 (9)	0.0684 (12)	−0.0046 (7)	−0.0104 (8)	0.0138 (8)
C21	0.0836 (16)	0.0532 (12)	0.0876 (16)	0.0003 (11)	−0.0119 (12)	0.0305 (11)
C13	0.0373 (9)	0.0401 (9)	0.0509 (10)	0.0046 (7)	−0.0028 (7)	0.0100 (7)
C14	0.0858 (17)	0.131 (2)	0.0647 (14)	0.0513 (16)	−0.0112 (12)	0.0354 (15)
N1	0.0286 (7)	0.0527 (8)	0.0573 (9)	0.0047 (6)	−0.0002 (6)	0.0176 (7)
O1	0.0374 (7)	0.0683 (9)	0.0731 (9)	0.0151 (6)	0.0019 (6)	0.0197 (7)
O2	0.0618 (9)	0.1045 (12)	0.0516 (8)	0.0386 (8)	0.0005 (6)	0.0289 (8)
S1	0.0402 (3)	0.0558 (3)	0.0645 (3)	−0.00397 (19)	0.0072 (2)	0.0190 (2)

### (II) Methyl 1-methyl-3-(o-tolyl)-1,2,3,3a,4,9b-hexahydrothiochromeno[4,3-b]pyrrole-3a-carboxylate Geometric parameters (Å, º)

**Table:**

C1—C6	1.389 (3)	C11—C12	1.540 (2)
C1—C2	1.394 (2)	C11—H11A	0.9700
C1—C9	1.506 (2)	C11—H11B	0.9700
C2—C3	1.377 (3)	C12—C15	1.505 (3)
C2—H2	0.9300	C12—H12	0.9800
C3—C4	1.372 (4)	C15—C16	1.391 (3)
C3—H3	0.9300	C15—C20	1.402 (2)
C4—C5	1.364 (3)	C16—C17	1.380 (3)
C4—H4	0.9300	C16—H14	0.9300
C5—C6	1.401 (3)	C17—C18	1.362 (4)
C5—H5	0.9300	C17—H15	0.9300
C6—S1	1.7495 (18)	C18—C19	1.362 (3)
C7—C8	1.522 (2)	C18—H16	0.9300
C7—S1	1.7967 (17)	C19—C20	1.397 (3)
C7—H7A	0.9700	C19—H17	0.9300
C7—H7B	0.9700	C20—C21	1.483 (3)
C8—C13	1.516 (2)	C21—H19A	0.9600
C8—C9	1.531 (2)	C21—H19B	0.9600
C8—C12	1.573 (2)	C21—H19C	0.9600
C9—N1	1.470 (2)	C13—O1	1.192 (2)
C9—H9	0.9800	C13—O2	1.327 (2)
C10—N1	1.450 (2)	C14—O2	1.453 (2)
C10—H10A	0.9600	C14—H21A	0.9600
C10—H10B	0.9600	C14—H21B	0.9600
C10—H10C	0.9600	C14—H21C	0.9600
C11—N1	1.458 (2)		
			
C6—C1—C2	117.86 (17)	C12—C11—H11B	110.3
C6—C1—C9	123.26 (15)	H11A—C11—H11B	108.6
C2—C1—C9	118.87 (16)	C15—C12—C11	117.07 (16)
C3—C2—C1	122.0 (2)	C15—C12—C8	116.40 (14)
C3—C2—H2	119.0	C11—C12—C8	102.78 (13)
C1—C2—H2	119.0	C15—C12—H12	106.6
C4—C3—C2	119.3 (2)	C11—C12—H12	106.6
C4—C3—H3	120.3	C8—C12—H12	106.6
C2—C3—H3	120.3	C16—C15—C20	118.02 (18)
C5—C4—C3	120.2 (2)	C16—C15—C12	121.01 (16)
C5—C4—H4	119.9	C20—C15—C12	120.96 (17)
C3—C4—H4	119.9	C17—C16—C15	122.0 (2)
C4—C5—C6	120.9 (2)	C17—C16—H14	119.0
C4—C5—H5	119.5	C15—C16—H14	119.0
C6—C5—H5	119.5	C18—C17—C16	119.5 (2)
C1—C6—C5	119.60 (18)	C18—C17—H15	120.2
C1—C6—S1	124.27 (13)	C16—C17—H15	120.2
C5—C6—S1	116.06 (15)	C19—C18—C17	119.9 (2)
C8—C7—S1	113.57 (12)	C19—C18—H16	120.1
C8—C7—H7A	108.9	C17—C18—H16	120.1
S1—C7—H7A	108.9	C18—C19—C20	122.1 (2)
C8—C7—H7B	108.9	C18—C19—H17	119.0
S1—C7—H7B	108.9	C20—C19—H17	119.0
H7A—C7—H7B	107.7	C19—C20—C15	118.43 (19)
C13—C8—C7	109.75 (13)	C19—C20—C21	118.35 (18)
C13—C8—C9	115.80 (13)	C15—C20—C21	123.19 (19)
C7—C8—C9	110.26 (13)	C20—C21—H19A	109.5
C13—C8—C12	109.12 (13)	C20—C21—H19B	109.5
C7—C8—C12	111.39 (14)	H19A—C21—H19B	109.5
C9—C8—C12	100.21 (12)	C20—C21—H19C	109.5
N1—C9—C1	113.43 (13)	H19A—C21—H19C	109.5
N1—C9—C8	101.16 (13)	H19B—C21—H19C	109.5
C1—C9—C8	116.72 (13)	O1—C13—O2	122.96 (16)
N1—C9—H9	108.4	O1—C13—C8	124.25 (16)
C1—C9—H9	108.4	O2—C13—C8	112.71 (14)
C8—C9—H9	108.4	O2—C14—H21A	109.5
N1—C10—H10A	109.5	O2—C14—H21B	109.5
N1—C10—H10B	109.5	H21A—C14—H21B	109.5
H10A—C10—H10B	109.5	O2—C14—H21C	109.5
N1—C10—H10C	109.5	H21A—C14—H21C	109.5
H10A—C10—H10C	109.5	H21B—C14—H21C	109.5
H10B—C10—H10C	109.5	C10—N1—C11	110.79 (15)
N1—C11—C12	106.89 (14)	C10—N1—C9	114.21 (15)
N1—C11—H11A	110.3	C11—N1—C9	105.48 (13)
C12—C11—H11A	110.3	C13—O2—C14	115.82 (16)
N1—C11—H11B	110.3	C6—S1—C7	102.79 (8)
			
C6—C1—C2—C3	1.6 (3)	C11—C12—C15—C16	41.8 (3)
C9—C1—C2—C3	−179.75 (17)	C8—C12—C15—C16	−80.2 (2)
C1—C2—C3—C4	−1.4 (3)	C11—C12—C15—C20	−139.27 (18)
C2—C3—C4—C5	−0.1 (3)	C8—C12—C15—C20	98.65 (19)
C3—C4—C5—C6	1.3 (3)	C20—C15—C16—C17	−1.4 (3)
C2—C1—C6—C5	−0.4 (2)	C12—C15—C16—C17	177.5 (2)
C9—C1—C6—C5	−178.98 (15)	C15—C16—C17—C18	−0.9 (4)
C2—C1—C6—S1	176.50 (12)	C16—C17—C18—C19	2.1 (4)
C9—C1—C6—S1	−2.1 (2)	C17—C18—C19—C20	−1.0 (4)
C4—C5—C6—C1	−1.0 (3)	C18—C19—C20—C15	−1.3 (3)
C4—C5—C6—S1	−178.16 (16)	C18—C19—C20—C21	176.6 (2)
S1—C7—C8—C13	−66.15 (15)	C16—C15—C20—C19	2.5 (3)
S1—C7—C8—C9	62.56 (15)	C12—C15—C20—C19	−176.46 (17)
S1—C7—C8—C12	172.89 (10)	C16—C15—C20—C21	−175.41 (19)
C6—C1—C9—N1	−94.84 (18)	C12—C15—C20—C21	5.7 (3)
C2—C1—C9—N1	86.56 (18)	C7—C8—C13—O1	−34.2 (2)
C6—C1—C9—C8	22.2 (2)	C9—C8—C13—O1	−159.81 (16)
C2—C1—C9—C8	−156.42 (15)	C12—C8—C13—O1	88.1 (2)
C13—C8—C9—N1	−163.87 (13)	C7—C8—C13—O2	149.08 (15)
C7—C8—C9—N1	70.79 (15)	C9—C8—C13—O2	23.5 (2)
C12—C8—C9—N1	−46.69 (14)	C12—C8—C13—O2	−88.61 (17)
C13—C8—C9—C1	72.56 (18)	C12—C11—N1—C10	−149.04 (17)
C7—C8—C9—C1	−52.78 (18)	C12—C11—N1—C9	−25.0 (2)
C12—C8—C9—C1	−170.27 (13)	C1—C9—N1—C10	−66.85 (19)
N1—C11—C12—C15	−133.89 (17)	C8—C9—N1—C10	167.34 (14)
N1—C11—C12—C8	−5.0 (2)	C1—C9—N1—C11	171.26 (15)
C13—C8—C12—C15	−77.38 (18)	C8—C9—N1—C11	45.45 (17)
C7—C8—C12—C15	43.94 (19)	O1—C13—O2—C14	−1.3 (3)
C9—C8—C12—C15	160.58 (14)	C8—C13—O2—C14	175.43 (19)
C13—C8—C12—C11	153.30 (16)	C1—C6—S1—C7	10.31 (16)
C7—C8—C12—C11	−85.38 (18)	C5—C6—S1—C7	−172.73 (13)
C9—C8—C12—C11	31.27 (17)	C8—C7—S1—C6	−40.14 (13)

### (III) Methyl 1-methyl-3-(o-tolyl)-3,3a,4,9b-tetrahydro-1H-thiochromeno[4,3-c]isoxazole-3a-carboxylate Crystal data

**Table:**

C_20_H_21_NO_3_S	*F*(000) = 1504
*M**_r_* = 355.44	*D*_x_ = 1.320 Mg m^−^^3^
Orthorhombic, *P**b**c**a*	Mo *K*α radiation, λ = 0.71073 Å
Hall symbol: -P 2ac 2ab	Cell parameters from 2536 reflections
*a* = 11.2629 (11) Å	θ = 1.7–25.0°
*b* = 13.2117 (11) Å	µ = 0.20 mm^−^^1^
*c* = 24.041 (3) Å	*T* = 293 K
*V* = 3577.3 (6) Å^3^	Block, colourless
*Z* = 8	0.35 × 0.30 × 0.25 mm

### (III) Methyl 1-methyl-3-(o-tolyl)-3,3a,4,9b-tetrahydro-1H-thiochromeno[4,3-c]isoxazole-3a-carboxylate Data collection

**Table:**

Bruker SMART APEXII CCD diffractometer	3151 independent reflections
Radiation source: fine-focus sealed tube	2536 reflections with *I* > 2σ(*I*)
Graphite monochromator	*R*_int_ = 0.033
ω and φ scans	θ_max_ = 25.0°, θ_min_ = 1.7°
Absorption correction: multi-scan (*SADABS*; Bruker, 2008)	*h* = −13→13
*T*_min_ = 0.932, *T*_max_ = 0.951	*k* = −15→15
37913 measured reflections	*l* = −25→28

### (III) Methyl 1-methyl-3-(o-tolyl)-3,3a,4,9b-tetrahydro-1H-thiochromeno[4,3-c]isoxazole-3a-carboxylate Refinement

**Table:**

Refinement on *F*^2^	Primary atom site location: structure-invariant direct methods
Least-squares matrix: full	Secondary atom site location: difference Fourier map
*R*[*F*^2^ > 2σ(*F*^2^)] = 0.046	Hydrogen site location: inferred from neighbouring sites
*wR*(*F*^2^) = 0.111	H-atom parameters constrained
*S* = 1.12	*w* = 1/[σ^2^(*F*_o_^2^) + (0.0267*P*)^2^ + 3.6376*P*] where *P* = (*F*_o_^2^ + 2*F*_c_^2^)/3
3151 reflections	(Δ/σ)_max_ < 0.001
229 parameters	Δρ_max_ = 0.22 e Å^−^^3^
0 restraints	Δρ_min_ = −0.22 e Å^−^^3^

### (III) Methyl 1-methyl-3-(o-tolyl)-3,3a,4,9b-tetrahydro-1H-thiochromeno[4,3-c]isoxazole-3a-carboxylate Special details

**Table:**

Geometry. All e.s.d.'s (except the e.s.d. in the dihedral angle between two l.s. planes) are estimated using the full covariance matrix. The cell e.s.d.'s are taken into account individually in the estimation of e.s.d.'s in distances, angles and torsion angles; correlations between e.s.d.'s in cell parameters are only used when they are defined by crystal symmetry. An approximate (isotropic) treatment of cell e.s.d.'s is used for estimating e.s.d.'s involving l.s. planes.
Refinement. Refinement of *F*^2^ against ALL reflections. The weighted *R*-factor *wR* and goodness of fit *S* are based on *F*^2^, conventional *R*-factors *R* are based on *F*, with *F* set to zero for negative *F*^2^. The threshold expression of *F*^2^ > σ(*F*^2^) is used only for calculating *R*-factors(gt) *etc*. and is not relevant to the choice of reflections for refinement. *R*-factors based on *F*^2^ are statistically about twice as large as those based on *F*, and *R*- factors based on ALL data will be even larger.

### (III) Methyl 1-methyl-3-(o-tolyl)-3,3a,4,9b-tetrahydro-1H-thiochromeno[4,3-c]isoxazole-3a-carboxylate Fractional atomic coordinates and isotropic or equivalent isotropic displacement parameters (Å^2^)

**Table:**

	*x*	*y*	*z*	*U*_iso_*/*U*_eq_	
C6	0.1021 (2)	0.96898 (18)	0.15232 (10)	0.0414 (6)	
C5	0.1663 (3)	1.0558 (2)	0.16587 (11)	0.0554 (8)	
H2	0.2488	1.0549	0.1644	0.067*	
C4	0.1082 (4)	1.1428 (2)	0.18133 (13)	0.0682 (9)	
H3	0.1516	1.2001	0.1908	0.082*	
C3	−0.0133 (4)	1.1458 (2)	0.18285 (13)	0.0661 (9)	
H4	−0.0524	1.2051	0.1928	0.079*	
C2	−0.0774 (3)	1.0602 (2)	0.16959 (11)	0.0532 (7)	
H5	−0.1599	1.0627	0.1704	0.064*	
C1	−0.0216 (2)	0.96998 (17)	0.15496 (9)	0.0387 (6)	
C9	−0.0973 (2)	0.87834 (17)	0.14347 (9)	0.0349 (5)	
H7	−0.1701	0.8994	0.1244	0.042*	
C8	−0.0406 (2)	0.79039 (17)	0.11168 (9)	0.0339 (5)	
C7	0.0803 (2)	0.76400 (18)	0.13613 (10)	0.0396 (6)	
H9A	0.1108	0.7038	0.1179	0.048*	
H9B	0.0713	0.7489	0.1754	0.048*	
C12	−0.0283 (2)	0.80429 (18)	0.04923 (10)	0.0402 (6)	
C13	−0.1085 (4)	0.8784 (3)	−0.03165 (13)	0.1103 (17)	
H11A	−0.0351	0.9079	−0.0439	0.165*	
H11B	−0.1734	0.9211	−0.0425	0.165*	
H11C	−0.1179	0.8128	−0.0483	0.165*	
C11	−0.1353 (2)	0.70812 (18)	0.12471 (10)	0.0405 (6)	
H12	−0.2058	0.7209	0.1019	0.049*	
C15	−0.0373 (2)	0.5505 (2)	0.15990 (12)	0.0485 (7)	
H13	−0.0221	0.5846	0.1930	0.058*	
C16	0.0011 (3)	0.4518 (2)	0.15331 (15)	0.0626 (9)	
H14	0.0427	0.4197	0.1817	0.075*	
C17	−0.0226 (3)	0.4015 (2)	0.10480 (16)	0.0698 (10)	
H15	0.0041	0.3355	0.1000	0.084*	
C18	−0.0850 (3)	0.4478 (2)	0.06366 (14)	0.0638 (8)	
H16	−0.1015	0.4122	0.0312	0.077*	
C19	−0.1248 (2)	0.5466 (2)	0.06874 (12)	0.0483 (7)	
C20	−0.1961 (3)	0.5916 (3)	0.02205 (13)	0.0751 (10)	
H18A	−0.1985	0.5450	−0.0086	0.113*	
H18B	−0.1600	0.6538	0.0102	0.113*	
H18C	−0.2755	0.6048	0.0347	0.113*	
C14	−0.0983 (2)	0.59892 (18)	0.11741 (10)	0.0382 (6)	
C10	−0.2337 (3)	0.8711 (2)	0.22232 (12)	0.0608 (8)	
H20A	−0.2966	0.8737	0.1954	0.091*	
H20B	−0.2167	0.9382	0.2353	0.091*	
H20C	−0.2576	0.8295	0.2531	0.091*	
N1	−0.12874 (18)	0.82867 (15)	0.19689 (8)	0.0399 (5)	
O3	−0.1645 (2)	0.72567 (14)	0.18158 (8)	0.0649 (6)	
O1	0.04071 (18)	0.75906 (16)	0.02133 (8)	0.0600 (5)	
O2	−0.1069 (2)	0.86808 (17)	0.02891 (7)	0.0757 (7)	
S1	0.18494 (6)	0.86516 (5)	0.12793 (3)	0.04623 (19)	

### (III) Methyl 1-methyl-3-(o-tolyl)-3,3a,4,9b-tetrahydro-1H-thiochromeno[4,3-c]isoxazole-3a-carboxylate Atomic displacement parameters (Å^2^)

**Table:**

	*U*^11^	*U*^22^	*U*^33^	*U*^12^	*U*^13^	*U*^23^
C6	0.0534 (15)	0.0403 (14)	0.0303 (13)	−0.0046 (12)	−0.0061 (11)	0.0067 (11)
C5	0.0688 (19)	0.0493 (17)	0.0482 (16)	−0.0181 (15)	−0.0132 (14)	0.0071 (13)
C4	0.105 (3)	0.0420 (18)	0.0574 (19)	−0.0220 (18)	−0.0143 (19)	−0.0011 (14)
C3	0.105 (3)	0.0359 (16)	0.0576 (19)	0.0024 (17)	−0.0008 (18)	−0.0053 (14)
C2	0.0725 (19)	0.0397 (15)	0.0472 (16)	0.0070 (14)	−0.0027 (14)	0.0022 (12)
C1	0.0539 (15)	0.0339 (13)	0.0282 (12)	0.0021 (11)	−0.0056 (11)	0.0023 (10)
C9	0.0377 (12)	0.0378 (13)	0.0293 (12)	0.0064 (10)	−0.0032 (10)	0.0017 (10)
C8	0.0361 (12)	0.0333 (12)	0.0325 (12)	0.0029 (10)	0.0010 (10)	−0.0002 (10)
C7	0.0386 (13)	0.0366 (13)	0.0436 (14)	0.0013 (11)	−0.0005 (11)	0.0016 (11)
C12	0.0456 (14)	0.0384 (14)	0.0364 (13)	−0.0014 (12)	0.0023 (12)	−0.0044 (11)
C13	0.171 (4)	0.129 (3)	0.0311 (17)	0.072 (3)	0.005 (2)	0.0142 (19)
C11	0.0380 (13)	0.0393 (13)	0.0441 (14)	0.0005 (11)	0.0040 (11)	−0.0018 (11)
C15	0.0466 (15)	0.0459 (16)	0.0531 (17)	−0.0084 (13)	−0.0029 (13)	0.0046 (13)
C16	0.0490 (16)	0.0498 (18)	0.089 (2)	0.0048 (14)	0.0027 (16)	0.0217 (17)
C17	0.067 (2)	0.0392 (16)	0.103 (3)	0.0065 (15)	0.025 (2)	−0.0020 (18)
C18	0.072 (2)	0.0492 (17)	0.070 (2)	−0.0109 (16)	0.0155 (17)	−0.0181 (16)
C19	0.0443 (15)	0.0470 (15)	0.0536 (17)	−0.0096 (12)	0.0025 (13)	−0.0044 (13)
C20	0.087 (2)	0.074 (2)	0.064 (2)	−0.0162 (19)	−0.0271 (19)	−0.0084 (17)
C14	0.0329 (12)	0.0365 (13)	0.0451 (14)	−0.0043 (10)	0.0030 (11)	−0.0009 (11)
C10	0.0687 (19)	0.0627 (19)	0.0510 (16)	0.0175 (16)	0.0203 (15)	0.0000 (14)
N1	0.0433 (11)	0.0410 (12)	0.0355 (11)	0.0055 (9)	0.0023 (9)	0.0018 (9)
O3	0.0916 (16)	0.0424 (11)	0.0607 (13)	−0.0102 (10)	0.0396 (12)	−0.0064 (9)
O1	0.0646 (12)	0.0726 (13)	0.0428 (11)	0.0123 (11)	0.0081 (10)	−0.0140 (10)
O2	0.1102 (18)	0.0868 (16)	0.0301 (10)	0.0516 (14)	0.0040 (11)	0.0056 (10)
S1	0.0383 (3)	0.0485 (4)	0.0519 (4)	−0.0047 (3)	−0.0019 (3)	0.0028 (3)

### (III) Methyl 1-methyl-3-(o-tolyl)-3,3a,4,9b-tetrahydro-1H-thiochromeno[4,3-c]isoxazole-3a-carboxylate Geometric parameters (Å, º)

**Table:**

C6—C5	1.395 (4)	C13—H11B	0.9600
C6—C1	1.395 (4)	C13—H11C	0.9600
C6—S1	1.759 (3)	C11—O3	1.425 (3)
C5—C4	1.374 (4)	C11—C14	1.512 (3)
C5—H2	0.9300	C11—H12	0.9800
C4—C3	1.369 (5)	C15—C16	1.383 (4)
C4—H3	0.9300	C15—C14	1.387 (4)
C3—C2	1.379 (4)	C15—H13	0.9300
C3—H4	0.9300	C16—C17	1.368 (5)
C2—C1	1.392 (4)	C16—H14	0.9300
C2—H5	0.9300	C17—C18	1.359 (5)
C1—C9	1.506 (3)	C17—H15	0.9300
C9—N1	1.485 (3)	C18—C19	1.385 (4)
C9—C8	1.531 (3)	C18—H16	0.9300
C9—H7	0.9800	C19—C14	1.392 (4)
C8—C12	1.519 (3)	C19—C20	1.503 (4)
C8—C7	1.523 (3)	C20—H18A	0.9600
C8—C11	1.555 (3)	C20—H18B	0.9600
C7—S1	1.793 (2)	C20—H18C	0.9600
C7—H9A	0.9700	C10—N1	1.444 (3)
C7—H9B	0.9700	C10—H20A	0.9600
C12—O1	1.188 (3)	C10—H20B	0.9600
C12—O2	1.317 (3)	C10—H20C	0.9600
C13—O2	1.462 (3)	N1—O3	1.466 (3)
C13—H11A	0.9600		
			
C5—C6—C1	119.9 (3)	H11B—C13—H11C	109.5
C5—C6—S1	116.4 (2)	O3—C11—C14	109.3 (2)
C1—C6—S1	123.51 (19)	O3—C11—C8	103.74 (19)
C4—C5—C6	120.3 (3)	C14—C11—C8	117.02 (19)
C4—C5—H2	119.9	O3—C11—H12	108.8
C6—C5—H2	119.9	C14—C11—H12	108.8
C3—C4—C5	120.5 (3)	C8—C11—H12	108.8
C3—C4—H3	119.8	C16—C15—C14	120.4 (3)
C5—C4—H3	119.8	C16—C15—H13	119.8
C4—C3—C2	119.5 (3)	C14—C15—H13	119.8
C4—C3—H4	120.2	C17—C16—C15	119.6 (3)
C2—C3—H4	120.2	C17—C16—H14	120.2
C3—C2—C1	121.6 (3)	C15—C16—H14	120.2
C3—C2—H5	119.2	C18—C17—C16	120.2 (3)
C1—C2—H5	119.2	C18—C17—H15	119.9
C2—C1—C6	118.1 (2)	C16—C17—H15	119.9
C2—C1—C9	118.6 (2)	C17—C18—C19	121.8 (3)
C6—C1—C9	123.3 (2)	C17—C18—H16	119.1
N1—C9—C1	109.36 (18)	C19—C18—H16	119.1
N1—C9—C8	101.30 (18)	C18—C19—C14	118.2 (3)
C1—C9—C8	117.7 (2)	C18—C19—C20	118.7 (3)
N1—C9—H7	109.3	C14—C19—C20	123.1 (3)
C1—C9—H7	109.3	C19—C20—H18A	109.5
C8—C9—H7	109.3	C19—C20—H18B	109.5
C12—C8—C7	109.1 (2)	H18A—C20—H18B	109.5
C12—C8—C9	116.09 (19)	C19—C20—H18C	109.5
C7—C8—C9	110.75 (19)	H18A—C20—H18C	109.5
C12—C8—C11	110.23 (19)	H18B—C20—H18C	109.5
C7—C8—C11	112.07 (19)	C15—C14—C19	119.7 (2)
C9—C8—C11	98.24 (18)	C15—C14—C11	119.4 (2)
C8—C7—S1	111.98 (16)	C19—C14—C11	120.8 (2)
C8—C7—H9A	109.2	N1—C10—H20A	109.5
S1—C7—H9A	109.2	N1—C10—H20B	109.5
C8—C7—H9B	109.2	H20A—C10—H20B	109.5
S1—C7—H9B	109.2	N1—C10—H20C	109.5
H9A—C7—H9B	107.9	H20A—C10—H20C	109.5
O1—C12—O2	123.6 (2)	H20B—C10—H20C	109.5
O1—C12—C8	123.8 (2)	C10—N1—O3	104.0 (2)
O2—C12—C8	112.5 (2)	C10—N1—C9	112.9 (2)
O2—C13—H11A	109.5	O3—N1—C9	104.98 (17)
O2—C13—H11B	109.5	C11—O3—N1	109.19 (17)
H11A—C13—H11B	109.5	C12—O2—C13	115.9 (2)
O2—C13—H11C	109.5	C6—S1—C7	101.32 (12)
H11A—C13—H11C	109.5		
			
C1—C6—C5—C4	−0.6 (4)	C9—C8—C11—O3	−40.3 (2)
S1—C6—C5—C4	175.2 (2)	C12—C8—C11—C14	77.5 (3)
C6—C5—C4—C3	−0.9 (5)	C7—C8—C11—C14	−44.3 (3)
C5—C4—C3—C2	1.0 (5)	C9—C8—C11—C14	−160.7 (2)
C4—C3—C2—C1	0.5 (5)	C14—C15—C16—C17	−0.7 (4)
C3—C2—C1—C6	−2.0 (4)	C15—C16—C17—C18	−1.0 (5)
C3—C2—C1—C9	177.3 (2)	C16—C17—C18—C19	1.1 (5)
C5—C6—C1—C2	2.0 (4)	C17—C18—C19—C14	0.6 (4)
S1—C6—C1—C2	−173.48 (18)	C17—C18—C19—C20	−178.4 (3)
C5—C6—C1—C9	−177.2 (2)	C16—C15—C14—C19	2.3 (4)
S1—C6—C1—C9	7.3 (3)	C16—C15—C14—C11	−177.7 (2)
C2—C1—C9—N1	−82.6 (3)	C18—C19—C14—C15	−2.2 (4)
C6—C1—C9—N1	96.7 (3)	C20—C19—C14—C15	176.6 (3)
C2—C1—C9—C8	162.6 (2)	C18—C19—C14—C11	177.8 (2)
C6—C1—C9—C8	−18.1 (3)	C20—C19—C14—C11	−3.3 (4)
N1—C9—C8—C12	163.35 (19)	O3—C11—C14—C15	−34.3 (3)
C1—C9—C8—C12	−77.5 (3)	C8—C11—C14—C15	83.1 (3)
N1—C9—C8—C7	−71.5 (2)	O3—C11—C14—C19	145.6 (2)
C1—C9—C8—C7	47.7 (3)	C8—C11—C14—C19	−96.9 (3)
N1—C9—C8—C11	46.0 (2)	C1—C9—N1—C10	86.3 (3)
C1—C9—C8—C11	165.10 (19)	C8—C9—N1—C10	−148.7 (2)
C12—C8—C7—S1	64.2 (2)	C1—C9—N1—O3	−161.01 (19)
C9—C8—C7—S1	−64.8 (2)	C8—C9—N1—O3	−36.0 (2)
C11—C8—C7—S1	−173.41 (16)	C14—C11—O3—N1	145.05 (19)
C7—C8—C12—O1	32.1 (3)	C8—C11—O3—N1	19.5 (2)
C9—C8—C12—O1	158.1 (2)	C10—N1—O3—C11	129.1 (2)
C11—C8—C12—O1	−91.4 (3)	C9—N1—O3—C11	10.3 (2)
C7—C8—C12—O2	−151.3 (2)	O1—C12—O2—C13	2.2 (5)
C9—C8—C12—O2	−25.3 (3)	C8—C12—O2—C13	−174.4 (3)
C11—C8—C12—O2	85.2 (3)	C5—C6—S1—C7	163.25 (19)
C12—C8—C11—O3	−162.1 (2)	C1—C6—S1—C7	−21.1 (2)
C7—C8—C11—O3	76.1 (2)	C8—C7—S1—C6	48.96 (19)
